# Mosquito mass rearing: who’s eating the eggs?

**DOI:** 10.1051/parasite/2019075

**Published:** 2019-12-20

**Authors:** Hanano Yamada, Carina Kraupa, Charles Lienhard, Andrew Gordon Parker, Hamidou Maiga, Danilo de Oliveira Carvalho, Minlin Zheng, Thomas Wallner, Jeremy Bouyer

**Affiliations:** 1 Insect Pest Control Laboratory, Joint FAO/IAEA Division of Nuclear Techniques in Food and Agriculture, International Atomic Energy Agency, Vienna International Centre P.O. Box 100 1400 Vienna Austria; 2 Museum of Natural History, Arthropoda Department C.P. 6434 CH-1211 Geneva 6 Switzerland; 3 Beneficial Insects Institute, Fujian Agriculture & Forestry University Fuzhou Fujian Province 350002 PR China

**Keywords:** *Liposcelis bostrychophila*, psocids, sterile insect technique, SIT, egg storage, *Aedes*

## Abstract

For the sterile insect technique, and other related biological control methods where large numbers of the target mosquito are reared artificially, production efficiency is key for the economic viability of the technique. Rearing success begins with high quality eggs. Excess eggs are often stockpiled and stored for longer periods of time. Any pests that prey on these eggs are detrimental to stockpiles and need to be avoided. Psocids of the genus *Liposcelis* (Psocoptera, Liposcelididae) are common scavengers consuming various types of organic material that are distributed globally and thrive in warm damp environments, making insectaries ideal habitats. In this short report, we investigated the species that has been found scavenging stored mosquito eggs in our insectary and identified it to be *Liposcelis bostrychophila* Badonnel, 1931. Additional observations were made to determine whether these predators indeed feed on mosquito eggs, and to suggest simple, effective ways of avoiding infestation.

## Abstract (fr)

Pour la technique des insectes stériles et les autres méthodes de lutte biologique associées, dans lesquelles un grand nombre de moustiques cibles sont élevés artificiellement, l’efficacité de la production est essentielle pour la viabilité économique de la technique. Le succès de l’élevage commence par des œufs de bonne qualité. Les œufs excédentaires sont souvent stockés pendant de longues périodes. Tous les organismes nuisibles qui exploitent ces œufs nuisent à ces stocks et doivent être évités. Les psoques du genre *Liposcelis* (Psocoptera, Liposcelididae) sont des charognards répandus qui consomment diverses matières organiques, sont répartis dans le monde entier et prospèrent dans des environnements chauds et humides, ce qui rend les insectariums des habitats idéaux pour eux. Dans ce court rapport, nous avons étudié l’espèce qui mangeait des œufs de moustiques stockés dans notre insectarium et nous avons déterminé qu’il s’agissait de *Liposcelis bostrychophila* Badonnel, 1931. D’autres observations ont été faites pour déterminer si ces prédateurs se nourrissent effectivement des œufs de moustiques et suggérer des moyens simples et efficaces pour éviter l’infestation.

## Introduction

Most people would probably wonder why nuisance insects like mosquitoes should ever be reared. However, the rearing of mosquitoes is important for species characterisation, retaining of reference specimens, assessments of repellent and insecticide efficacy and/or resistance, and other research. In fact, several medically important mosquito species are mass reared for their deployment in the framework of the sterile insect technique (SIT), and other related biological control methods [5, 10]. The SIT relies on the mass production of reproductively sterilised male insects, which are released into a target area to compete for, and mate with, wild females, who subsequently lay infertile eggs gradually leading to a decline in the natural population of that particular pest species [10].

The mass rearing of millions of mosquitoes can be a costly undertaking and therefore production efficiency is crucial at all steps of the process, beginning with egg production and egg hatch. Mosquito eggs, such as those of some *Aedes* spp. (including potential vectors of dengue, chikungunya, yellow fever, Zika, and more) require a substrate for oviposition (usually crepe, or germination paper), drying and storage for several days for embryo maturation before they can be hatched. Excess eggs are also stockpiled for production security.

**Figure 1:**
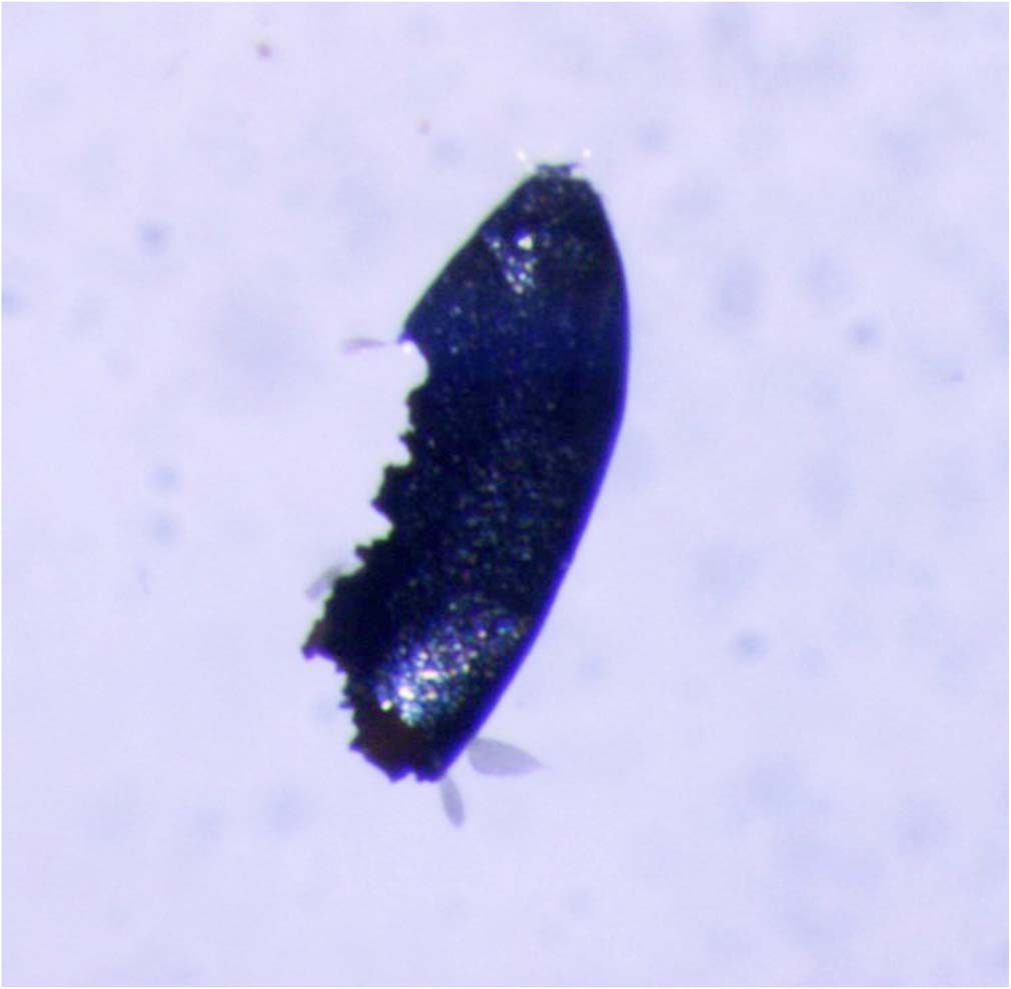
Mosquito egg showing severe damage. Photo by M. Zheng.

At the Insect Pest Control Laboratory of the Joint FAO/IAEA Division of Nuclear Techniques in Food and Agriculture, the Human Disease Vectors group is carrying out crucial research for the development of the SIT to manage mosquito vectors. Recent results include the cost reduction and optimisation of several components of the SIT package, including mass-rearing cages, larval diet, irradiation, handling, and quality control [3, 4, 8, 15, 16, 24]. The insectary currently stocks several mosquito strains for this research, and egg storage is an important component of the facility. Recently, it was discovered that some batches of mosquito eggs were hatching poorly, and the possible reasons for this were investigated. Some reasons for poor egg hatch in *Aedes* eggs are suboptimal storage conditions (conditions that are too dry desiccate the eggs making them collapse, killing the embryo; conditions that are too humid can trigger premature hatching, and embryos die shortly after) and mechanical damage of the eggs (mostly due to poor collection methods). However, it was found when checked under a stereomicroscope (Leica MZ 16 FA) that the eggs had been severely damaged and had the appearance of having been chewed by an unwanted guest (Fig. 1).

**Figure 2:**
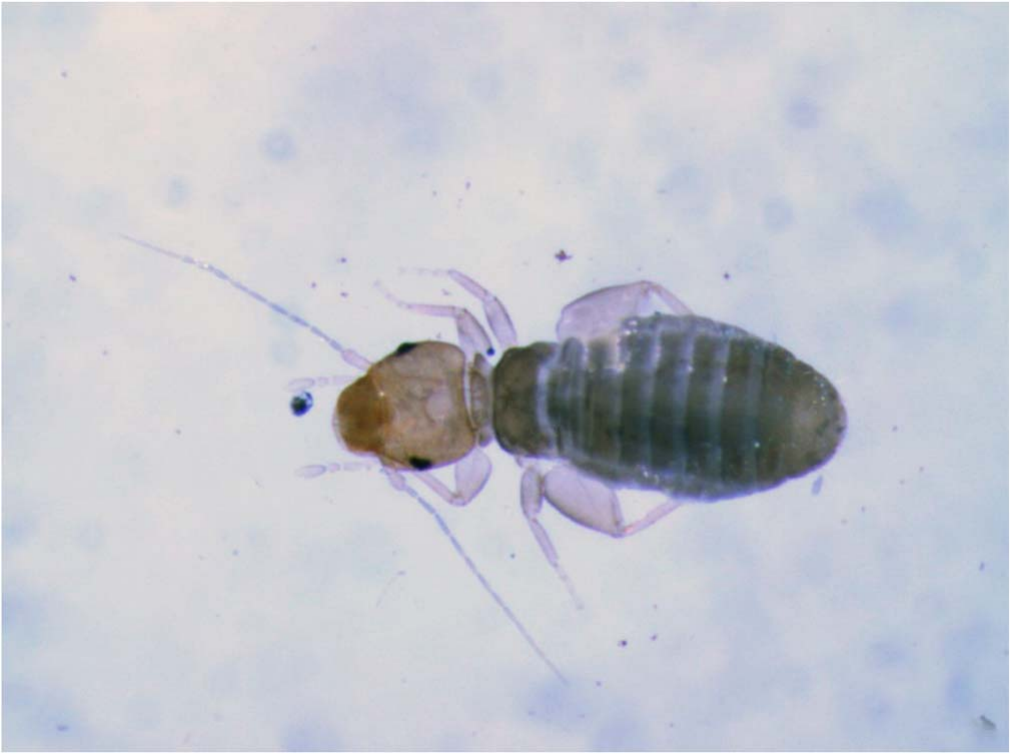
Psocid *Liposcelis bostrychophila* Badonnel, 1931 (Psocoptera, Liposcelididae) as seen on an egg paper. Photo by M. Zheng.

After a short search, the likely cause of the damaged eggs was detected crawling across the egg papers (Fig. 2). Communication with various laboratories rearing insects indicated that psocids have often been seen in insectaries, but there appears to be only one prior report about their appearance in a mosquito rearing environment [12]. Attempts to obtain the full text of this report were not successful.

Psocids of the genus *Liposcelis* (Psocoptera: Liposcelididae) are common scavengers that are distributed globally and are known for being a nuisance pest in households, libraries, warehouses and food stores [9, 11]. They are an important pest found to damage valuable books, documents, and museum specimens [6]. They thrive in warm damp environments, often scavenging stored products, fungi, dead insect parts, starch – essentially any organic matter – reflecting their scientific name which loosely means “insects that like to chew” (from the Greek verb psochein = chew). They are also known to prefer dark environments [13], making the black, damp egg storage boxes containing papers with insect parts and eggs a luxury all-inclusive habitat for these insects.

To gain an idea of their importance and possible damage potential in mosquito rearing insectaries, a small test was performed to verify these predators as the cause of egg destruction.

## Materials, methods and results

Specimens were collected using a small brush and preserved in 90% ethanol. Samples were sent to the Arthropoda Department of the Museum of Natural History, Geneva, Switzerland. They were identified as females and nymphs of *Liposcelis bostrychophila* Badonnel, 1913 [1] (Psocoptera: Liposcelididae) (Fig. 2). Two microscopical slides containing females and nymphs (identified by CL) are deposited in the Psocoptera collection of the Geneva Natural History Museum. The body length of the medium-brown females of this species is about 1 mm. All species of the genus *Liposcelis* are apterous (i.e. completely wingless) in both sexes and they are characterised by their dorso-ventrally flattened body and the strongly enlarged hind femora.

Second, to verify that the psocids were truly responsible for the reduced hatch rates and the damaged eggs, we transferred 10 *L. bostrychophila* (of mixed/unknown age) to each of 5 egg papers (i.e. 5 repetitions) holding 200–300 eggs and placed them in a petri dish and into the egg boxes for 12 weeks. Two egg papers from the same mosquito cohort but without psocids were kept as controls. The egg papers were then checked periodically using a stereomicroscope. Damaged eggs and numbers of psocids were recorded.

**Figure 3:**
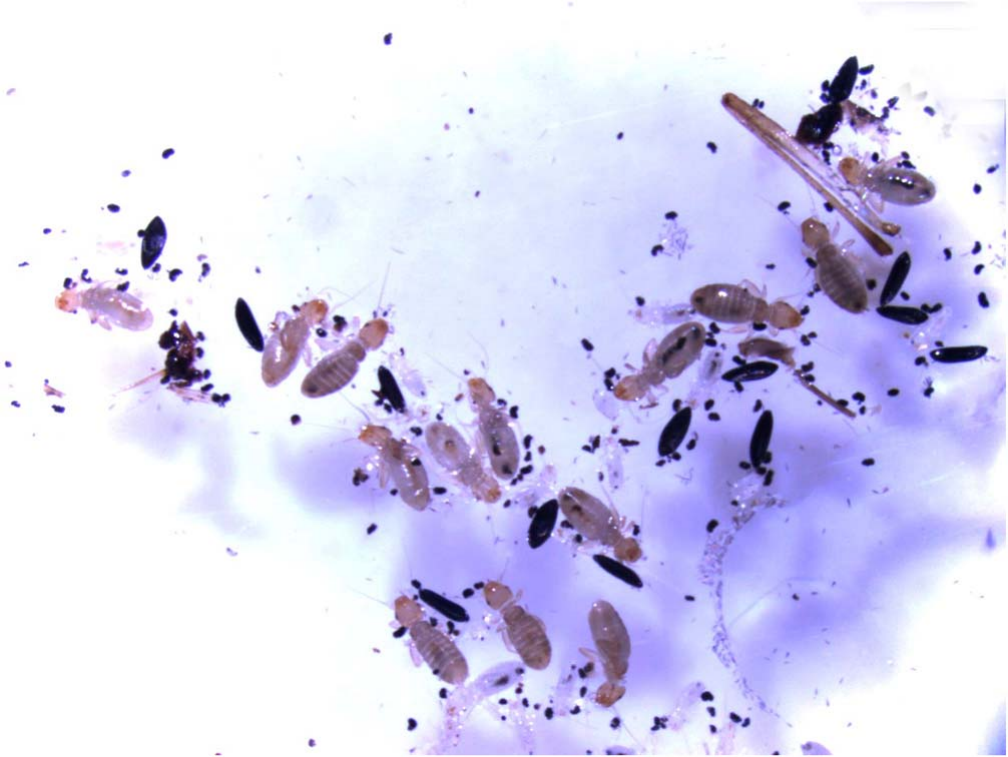
Treatment papers with heavy infestation (31 individual psocids and 14 intact eggs within the field of view). Photo by M. Zheng.

After three months, the contaminated egg papers were heavily infested with the *L. bostrychophila* (~100 individuals [103 ± 14]) while the control egg papers had none (Fig. 3). The number of damaged eggs was also significant (between 38.9% and 50.8%) compared to just a few collapsed eggs on the control papers (<3%). The difference in damage was clear: the eggs damaged by the psocids presented visible bite marks, and the psocids appeared to be fat and numerous.

**Figure 4:**
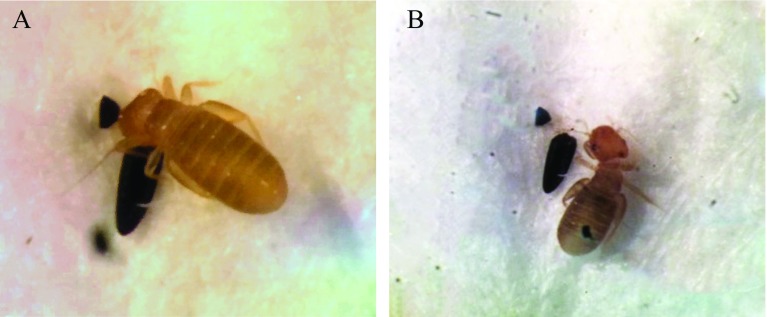
Before (A) and after (B) a psocid meal. Small pieces of the egg chorion seen in the insect’s abdomen. Photo by H. Yamada.

Psocids were also observed eating the egg chorions (Fig. 4A). Parts of the black egg can be observed inside the abdomen (Fig. 4B).

## Discussion

*Liposcelis bostrychophila* is a cosmopolitan species and is the most common psocid in domestic settings, food processing plants, and grain stores. A comprehensive review on its biology, population dynamics and physiology was published by Turner [20]. In particular, a case of egg predation in an anobiid beetle by *L. bostrychophila* was studied in detail by Williams [23] and predation on Indian meal moth eggs (*Plodia interpunctella*) was recorded by Lovitt and Soderstrom [14]. The eggs of the cigarette beetle *Lasioderma serricorne* have also been seen to be preyed upon by *L. divinatoria* [19]. Thus, the predation on the eggs of other insects is not unusual for some *Liposcelis* spp, but the significance thereof in insect rearing facilities has not been studied, or reported to date. The species is usually parthenogenetic (thelytokous), can diapause, is facultatively cannibalistic, and can survive without food for up to 2 months [21]. For these reasons, these psocids are hard to eradicate once established in the insectary.

The problem in the insectary is obvious and the egg damage can be considerable if psocid infestation is not kept under control. It seems that they only feed on the egg chorion and not the embryo inside. However, damaging the chorion leads to embryo mortality nonetheless, due to dehydration. In addition to this, they may cause a health issue such as asthmatic reactions and allergies in insectary staff as they transfer microorganisms with their faeces and cast skin [7, 18, 22].

The psocids also seem to feed on the fungi that sometimes build up around dead adult mosquito parts that may be left on the egg papers. We have caught psocids in the packages of oviposition paper from suppliers, and they become established in the cracks and creases of laboratory furniture (especially when made of plywood). Cosy, warm and damp mosquito rearing rooms seem to be a perfect environment for these insects and they are difficult to eradicate once established. Museums and libraries encounter similar problems with psocid infestations in books and other important documents and specimens. Their approaches to eliminating psocids include fumigation with methyl bromide, phosphine or ethylene dibromide, pyrethroid and carbamate insecticides, or mixtures with piperonyl butoxide, or the placement of naphthalene balls [6, 13, 17]. However, these insects are rapidly becoming resistant to such chemical treatments. In the case of insectaries or mass rearing facilities, most chemicals including essentially all insecticides are banned for obvious reasons. Heat treatments have also been used [2].

We advise the following procedures to prevent egg loss due to predation by psocids: (i) maintain a clean insectary to prevent psocids from entering the insectary on various paper products or cardboard boxes to begin with; (ii) oviposition papers (seed germination papers) that are used for mosquito oviposition should be frozen (100% mortality is achieved at 0 °C [13]), or heated in the microwave (mortality above 35 °C [25]) before entering the laboratories and before use; (iii) egg storage boxes should be purged with boiling water routinely to ensure the elimination of any psocids and their eggs. These simple preventive practices were efficient to ensure minimal egg loss and to keep psocid numbers in check.

## Competing interests

The authors declare that they have no competing interests.

## References

[1] Badonnel A. 1931 Contribution à l'étude de la faune du Mozambique. Voyage de M. P. Lesne (1928–1929). 4e note. Copéognathes. Annales des Sciences Naturelles, Zoologie, 10(14), 229–260.

[2] Beckett S, Morton R. 2003 The mortality of three species of Psocoptera*, Liposcelis bostrychophila* Badonnel, *Liposcelis decolor* Pearman and *Liposcelis paeta* Pearman, at moderately elevated temperatures. Journal of Stored Products Research, 39, 103–115.

[3] Bimbilé Somda NS, Dabiré KR, Maiga H, Yamada H, Mamai W, Gnankiné O, Diabaté A, Sanon A, Bouyer J, Gilles JRL. 2017 Cost-effective larval diet mixtures for mass rearing of *Anopheles arabiensis* Patton (Diptera: Culicidae). Parasites & Vectors, 10, 619. 29273056 10.1186/s13071-017-2552-3 PMC5741881

[4] Bimbilé Somda NS, Maïga H, Mamai W, Yamada H, Ali A, Konzcal A, Gnankine O, Diabate A, Sanon A, Dabire KR, Gilles JRL, Bouyer J. 2019 Insects to feed insects – feeding *Aedes* mosquitoes with flies for laboratory rearing. Scientific Reports, 9(1), 11403. 31388041 10.1038/s41598-019-47817-x PMC6684809

[5] Bourtzis K, Lees RS, Hendrichs J, Vreysen MJB. 2016 More than one rabbit out of the hat: Radiation, transgenic and symbiont-based approaches for sustainable management of mosquito and tsetse fly populations. Acta Tropica, 157, 115–130. 26774684 10.1016/j.actatropica.2016.01.009

[6] Chin HC, Jeffery J, Ahmad NW, Kiang HS. 2010 First report of *Liposcelis bostrychophila* Badonnel (Psocoptera: Liposcelidae) as a museum insect pest in Malaysia. Sains Malaysiana, 39, 329–331.

[7] Clemmons EA, Taylor DK. 2016 Booklice (*Liposcelis* spp.), grain mites (*Acarus siro*), and flour beetles (*Tribolium* spp.): ‘Other pests’ occasionally found in laboratory animal facilities. Journal of the American Association for Laboratory Animal Science, 55, 737–743. 27931310 PMC5113873

[8] Culbert NJ, Balestrino F, Dor A, Herranz GS, Yamada H, Wallner T, Bouyer J. 2018 A rapid quality control test to foster the development of genetic control in mosquitoes. Scientific Reports, 8, 16179. 30385841 10.1038/s41598-018-34469-6 PMC6212531

[9] Diaz-Montano J, Campbell JF, Phillips TW, Throne JE. 2014 Evaluation of potential attractants for *Liposcelis bostrychophila* (Psocoptera: Liposcelididae). Journal of Economic Entomology, 107, 867–874. 24772572 10.1603/ec13427

[10] Dyck VA, Hendrichs JP, Robinson AS. 2005 The sterile insect technique: Principles and practice in area-wide integrated pest management. Springer: Dordrecht.

[11] Gautam SG, Opit GP, Shakya K. 2016 Population growth and development of the psocid *Liposcelis fusciceps* (Psocoptera: Liposcelididae) at constant temperatures and relative humidities. Environmental Entomology, 45, 237–244. 26385931 10.1093/ee/nvv148

[12] Gerberg EJ. 1961 Quarterly report no. 3, 1 July-30 Sep 61. U.S. Government Research Reports, 36, 70.

[13] Green PWC, Turner BD. 2005 Food-selection by the booklouse, *Liposcelis bostrychophila* Badonnel (*Psocoptera: Liposcelididae*). Journal of Stored Products Research, 41, 103–113.

[14] Lovitt AE, Soderstrom EL. 1968 Predation on Indian meal moth eggs by *Liposcelis bostrychophilus*. Journal of Economic Entomology, 61, 1444–1445.

[15] Maiga H, Bimbilé-Somda NS, Yamada H, Wood O, Damiens D, Mamai W, Balestrino F, Lees RS, Dabiré RK, Diabaté A, Gilles JRL. 2017 Enhancements to the mass-rearing cage for the malaria vector, Anopheles arabiensis for improved adult longevity and egg production. Entomologia Experimentalis et Applicata, 164, 269–275.

[16] Maïga H, Mamai W, Bimbilé Somda NS, Konczal A, Wallner T, Herranz GS, Herrero RA, Yamada H, Bouyer J. 2019 Reducing the cost and assessing the performance of a novel adult mass-rearing cage for the dengue, chikungunya, yellow fever and Zika vector, *Aedes aegypti* (Linnaeus). PLoS Neglected Tropical Diseases, 13(9), e0007775. 31553724 10.1371/journal.pntd.0007775 PMC6779276

[17] Nayak M. 2006 Psocid and mite pests of stored commodities: small but formidable enemies, in Proc. 9th Int. Work. Conf. Stored Prod. Prot. Brazilian Post-harvest Association-ABRAPOS, Campinas, Brazil, pp. 1061–1073.

[18] Opit G, Ocran A, Shakya K. 2018 Population growth and development of *Liposcelis obscurus* Broadhead (Psocodea: Liposcelididae) at constant temperatures and relative humidities. Julius-Kuehn-Archiv, 463, 151–159.

[19] Rao ChV, Rao N, Narasimha B, Ramesh BT. 2002 New records of predation on the eggs of cigarette beetle, *Lasioderma serricorne* (Fabricius) (Coleoptera: Anobiidae), a stored tobacco pest. Journal of Biological Control, 16(2), 169–170.

[20] Turner BD. 1994 *Liposcelis bostrychophila* (Psocoptera: Liposcelididae), a stored food pest in the UK. International Journal of Pest Management, 40, 179–190.

[21] Turner BD, Maude-Roxby H. 1988 Starvation survival of the stored product pest *Liposcelis bostrychophilus* Badonnel (Psocoptera: Liposcelididae). Journal of Stored Product Research, 24, 23–28.

[22] Turner BD, Staines N, Brostoff J, Howe CA, Cooper K. 1996 Allergy to psocids in the UK, inProceedings of the 2nd International Conference on Insect Pests in the Urban Environment, Edinburgh, Wildey KB, Editor. p. 609

[23] Williams LH. 1972 Anobiid beetle eggs consumed by a psocid (Psocoptera: Liposcelidae). Annals of the Entomological Society of America, 65(533), 536.

[24] Yamada H, Maiga H, Juarez J, De Oliveira Carvalho D, Mamai W, Ali A, Bimbile-Somda NS, Parker AG, Zhang D, Bouyer J. 2019 Identification of critical factors that significantly affect the dose-response in mosquitoes irradiated as pupae. Parasites & Vectors, 12, 435. 31500662 10.1186/s13071-019-3698-y PMC6734225

[25] Yusuf M, Turner B. 2004 Characterisation of *Wolbachia*-like bacteria isolated from the parthenogenetic stored-product pest psocid *Liposcelis bostrychophila* (Badonnel) (Psocoptera). Journal of Stored Products Research, 40, 207–225.

